# Taxonomic profiling of skin microbiome and correlation with clinical skin parameters in healthy Koreans

**DOI:** 10.1038/s41598-021-95734-9

**Published:** 2021-08-11

**Authors:** Ji-Hee Kim, Sang-Mo Son, Hyunjoon Park, Byoung Kook Kim, In Suk Choi, Heebal Kim, Chul Sung Huh

**Affiliations:** 1 WCU Biomodulation Major, Department of Agricultural Biotechnology, College of Agriculture and Life Sciences, Seoul National University, Seoul, 08826 South Korea; 2 Research Institute of Eco-Friendly Livestock Science, Institute of Green-Bio Science and Technology, Pyeongchang-gun, Gangwon-do 25354 South Korea; 3 Chong Kun Dang Bio Research Institute, Ansan-si, Gyeonggi-do 15604 South Korea; 4 Department of Agricultural Biotechnology and Research Institute of Agriculture and Life Sciences, Seoul National University, Seoul, 08826 South Korea; 5 Graduate School of International Agricultural Technology, Seoul National University, Pyeongchang-gun, Gangwon-do 25354 South Korea

**Keywords:** Computational biology and bioinformatics, Microbiology, Physiology, Medical research

## Abstract

The interest in skin microbiome differences by ethnicity, age, and gender is increasing. Compared to other ethnic groups, studies on the skin microbiome of Koreans remains insufficient; we investigated facial skin microbiome characteristics according to gender and age among Koreans. Fifty-one healthy participants were recruited, the facial skin characteristics of each donor were investigated, their skin bacterial DNA was isolated and metagenomic analysis was performed. The donors were divided into two groups for age and sex each to analyze their skin microbiomes. Moreover, we investigated the correlation between the skin microbiome and clinical characteristics. The alpha diversity of the skin microbiome was significantly higher in the elderly, and beta diversity was significantly different according to age. The comparative skin microbials showed that the genus *Lawsonella* was more abundant in the younger age group, and *Enhydrobacter* was predominant in the older age group. *Staphylococcus* and *Corynebacterium* were more abundant in males, while *Lactobacillus* was more abundant in females. *Lawsonella* had a negative correlation with skin moisture and brown spots. *Staphylococcus* and *Corynebacterium* both had negative correlations with the number of UV spots and positive correlations with transepidermal water loss (TEWL). Furthermore, *Staphylococcus aureus* had a negative correlation with skin moisture parameters.

## Introduction

The human skin is the largest organ, a complex and dynamic ecosystem inhabited by bacteria, fungi, and viruses^1^. The skin microorganisms and the human body have a symbiotic relationship to protect against invading pathogens, educate our immune system, and break down natural products^2–4^. In addition, various skin microorganism metabolites affect skin cells and exhibit a wide range of effects on skin barrier function, anti-aging, and anti-inflammatory^5,6^. Several studies clarifying the role of skin microorganisms in the skin through bacterial 16S ribosomal RNA gene sequencing have dramatically developed microbial identification technologies and provided insights into the improved microbial environment of the diverse ecosystem that were not previously understood^7^.

As the skin ages, structural changes occur in skin, and its functional characteristics change^8^. In modern society, interest in skincare and anti-aging is increasing and efforts are being made to find the cause of skin aging^9^. Several studies have been published on the association between the skin microbiome and skin aging^5,10^. It is known that alterations in the skin microbiome are accompanied by changes in individual skin conditions and physiology and phylogenetic diversity diminishes among aging individuals^10^.

The metabolites produced in the skin play an essential role in host- microorganism interactions and their production is greatly influenced by our environment and behavior^7,11^. In a study of 71 Chinese, skin microbiome changes were influenced by skin site, age, sex, and area of residence^12^. Furthermore, according to a recently published paper, skin microbiome in Korean women showed different patterns according to age, skin area, and occupation^13^. From these previous studies, we propose that the skin microbiome analysis in Koreans should not be limited to a specific gender. Paolo et al*.* suggested that understanding the physiological changes in the skin according to gender can help derive cosmetic improvement methods to prevent skin aging^14^.

In this study, we focused on the facial skin microbiome of Koreans to investigate the differences by age and gender. Taxonomic differences in skin microbiota were compared and analyzed between the younger and older age groups and between male and female groups. In addition, comparing and analyzing the skin microbiome and clinical information of healthy individuals was intended to lay a foundation for product development utilizing the skin microbiome.

## Methods

### Sample collection

Fifty-one healthy volunteers were recruited for collecting skin samples to investigate their skin microbiome characteristics. Recruitment was conducted only among those who signed written consent for the collection of material of human origin. The selection criteria were applied after IRB approval to collect human materials at Kyunghee University's Skin Biotechnology Center (IRB No. KHUSBC 2018-MB). All experiments were performed in accordance with relevant guidelines and regulations. The participants were divided into two age groups: 25 subjects in the younger group (“Young,” 21–36 years, mean age: 26.4 ± 3.8) and 26 subjects of the older group (“Old,” 49–67 years, mean age: 58.1 ± 4.8). Subjects were recruited regardless of gender; 25 males and 26 females. Before the sample collection, all participants washed their faces with the same facial cleanser that is a common type of foam cleanser that includes sodium lauryl sulfate (SLS) and samples were collected between 30 minutes and 1 hour after washing without any skin conditioner. Although we were aware that the skin microbiome could be affected by facial washing, we wanted to identify the skin microbial flora that are not washed out and remain constant on the skin surface even after facial washing. Skin samples were collected in five replicates per participant from two sites on the cheek and forehead, with sterile cotton swabs. To examine the overall microbial community for each facial skin area and reduce the bias between samples, we collected skin samples from five different areas of the forehead from each donor and performed the same procedure for the cheek. The swab tips were cut and transferred to collection tubes that contained 2 mL of 0.45% monopotassium phosphate (KH_2_PO_4_), 0.6% Disodium phosphate (Na_2_HPO_4_), and 0.05% L-cysteine HCl∙H_2_O, 0.05% Tween 80, immediately stored at 4℃ before DNA extraction.

### Clinical skin parameter measurement

Participants’ skin condition was investigated 30 min after facial washing for skin moisture, transepidermal water loss (TEWL), sebum level, skin texture (smoothing), periorbital wrinkle (average wrinkle depth, wrinkle volume, and wrinkle area), skin redness, moles, UV spots, brown spots, porphyrin, and skin tone^5^. Skin moisture was measured by Corneometer® (Courage + Khazaka electronic GmbH, Germany). TEWL was measured by Vapometer® (Delfin Technologies, Kuopio, Finland), and the skin sebum level was measured by Sebumeter® (Courage + Khazaka electronic GmbH, Germany). The skin texture and the periorbital wrinkles were measured by PRIMOS® (Canfield Scientific, USA). The skin redness was measured by a Spectrophotometer CM700d (Konica Minolta, Japan), and the measurements indicated a * value. All spot measurements and skin tones were measured by VISIA (Canfield Scientific, USA) skin analysis. All methods were carried out according to the equipment manufacturer's instructions.

### Genomic DNA extraction

The total bacterial gDNA was extracted from 510 samples collected at two different sites (cheek and forehead), conducted by a partially modified method using the QIAamp DNA Mini Kit (Qiagen, Germany, cat. no. 51306). Initially, the skin samples' collection tubes were dispensed with 20 μL Protease K, 540 μL Phosphate-buffered Saline (Corning, USA), and 60 μL AL buffer (Qiagen, Germany). After incubation at 56℃ for 3 hours, sample tubes were vortexed for 15 seconds. Then 600 μL of the AL buffer was added, vortexed for 15 seconds, and heat-treated at 56℃ for 10 minutes using a heat block. Six hundred microliters of 100% ethanol was added to the heat-treated sample, vortexed for 15 seconds, and 600 μL of the mixed sample was placed in the tube containing the spin column. After centrifugation at 14,000 rpm for one minute, the buffer that came out under the filter was discarded and the remaining sample was put back into the spin column. The above process was carried out for 510 samples, collected using the same spin column process for each facial skin area from ​​each donor to concentrate into 102 samples. The process was carried out following the manufacturer’s instructions (QIAamp DNA Mini and Blood Mini Handbook). The concentration and purity of the extracted genomic DNA were measured using a nanodrop (Thermo Scientific™, USA) according to the manufacturer's instructions.

### PCR amplification and 16S rRNA sequencing

The amplification of the V3–V4 region of the bacterial 16S rRNA gene was performed using barcoded 341F (5′-TCGTCGGCAGCGTC-AGATGTGTATAAGAGACAG-CCTACGGGNGGCWGCAG-3′) and 805R primer (5′-GTCTCGTGGGCTCGG-AGATGTGTATAAGAGACAG-GACTACHVGGGTATCTAATCC-3′) primers^15^. PCR conditions were performed as follows: initial denaturation at 95℃ for one min, 34 cycles of 95℃ for 30 s, 55℃ for 30 s, and 72℃ for 30 s, followed by a final extension at 72℃ for 5 min. The PCR-completed sample was cleaned using a PCR Purification Kit (Qiagen, Germany), and DNA concentration was confirmed using a nanodrop. The 16S rRNA gene library construction was carried out by Illumina's Demonstrated Protocol. After a quality check using Quanti-iT pICOgREEN dsDNA assay kit (Invitrogen), Illumina Miseq was performed under 500 + 7 cycles conditions using the MiSeq Reagent Kit (Illumina, San Diego, CA, USA) according to the manufacturer's instructions.

### Skin microbiome analysis

The 16S rRNA gene sequence was identified in EzBioCloud using the Microbiome Taxonomic Profiling (MTP) pipeline provided by ChunLab, Inc.^16^. Quality control of raw data was performed according to ChunLab in-house process^17,18^. Taxonomic assignments were acquired by the USEARCH tool, which searches and clusters algorithms that calculate sequence similarity against reads in the EzBioCloud database (https://www.ezbiocloud.net)^16^. The bacterial OTUs were identified by UCLUST, clustering of the 16S rRNA sequences with a ≥97% identity threshold, for taxonomic profiling analysis. Skin microbiome analysis based on the classification and identification of 16S rRNA sequences was performed using the MTP platform of the EzBioCloud database. Based on the OTUs data obtained through taxonomic profiling, differences in alpha- and beta-diversity between groups were analyzed using the R program (version 4.0.4, http://www.R-project.org/)^19^. The differences in relative abundance of skin microflora between groups were also confirmed using the same program. The R program was performed in RStudio (1.4.1), an integrated development environment.

### Statistical analysis

Statistical significance was demonstrated using the Wilcoxon signed-rank test to compare the two groups; the *P* values of the results are indicated in each chart. A comparative analysis of the clinical skin evaluation between the younger and older groups was conducted using the Student's t-test. Non-metric multidimensional scaling (NMDS) was applied to confirm the difference in distance between groups using the Multiple Response Permutation Procedure (“mrpp”) function^20^, of Vegan package (v 2.5-7) and “indval”, indicator species analysis^21^, function in “labdsv” package in R program. Permutational Multivariate Analysis of Variance was used to confirm the significance of beta diversity between groups. Linear discriminant analysis (LDA) effect size tool- (LEfSe) was utilized to identify genera with relative differential abundance between groups using the web-based application “Galaxy” version 1.0 (https://huttenhower.sph.harvard.edu/galaxy/)^22^. Pearson correlation analysis was conducted using correlation test in R program to verify the correlation coefficient and significance level within *P* < 0.05.

### Ethics declaration

The selection criteria were applied after the IRB approval to collect human materials at Kyunghee University's Skin Biotechnology Center (IRB No. KHUSBC 2018-MB).

### Approval for human experiments

The clinical experiment of this study was conducted by requesting the collection of human epidermal samples and participants’ clinical skin characteristics from Kyunghee University's Skin Biotechnology Center. Kyunghee University's Skin Biotechnology Center is an institution registered with the IRB at the Korea Centers for Disease Control and Prevention (No. 2-1040497-A-N-02), and all experiments were conducted after IRB approval (IRB No. KHUSBC 2018-MB). The 16S rRNA sequencing collected from human epidermal samples was carried out by ChunLab Inc. and skin characteristics were identified by Kyunghee University's Skin Biotechnology Center. Both institutions were ISO9001 certified and analyzed according to the appropriate regulations and guidelines for each investigating items. The clinical experiments were performed in accordance with the guidelines and regulations for clinical study of Korea Food and Drug Administration based on the Declaration of Helsinki.

### Consent to participate/consent to publish

Consent was obtained from all subjects to participate prior to conducting the study and posting consent was obtained from all clinical research participants. The consent for publication of articles was obtained from all authors.

## Results

### Skin characteristics

A total of 51 Koreans were examined for skin dermatological properties, all without skin-related diseases, none having used antibiotics or antifungal drugs within the past 3 months, and none had performed medical skincare within the past 6 months. Before sample collection, a clinical survey was conducted of each subject’s skin characteristics, which were compared by dividing participants into a younger and older group (Table 1). Additionally, we divided participants into males and females to determine whether the clinical characteristics of their skin differ by gender (Table 1). Significant differences were found between the younger and older group in the moisture content (cheek), periorbital wrinkles, and the number of spots (moles, UV spots, brown spots, and porphyrin). Moreover, the elderly group showed high values ​​in previously mentioned clinical parameters with significant differences. The clinical skin characteristics were compared by gender; there were significant differences in many skin parameters. The skin moisture content and skin tone were significantly higher in the female group; the number of UV spots and brown spots were higher in women. Simultaneously, oil content, transdermal moisture loss, average skin roughness, skin redness, amount of moles, and porphyrin levels were significantly higher in the male group.

**Table 1:** Skin characteristics of the subjects by age and gender.

Variables	Group		*P* value	Group		*P* value
	Young (n = 25)	Old (n = 26)		Male (n = 25)	Female (n = 26)	
Gender (male/female)	13/12	12/14	–	–	–	
Age (yr)	26.4 ± 3.8	58.1 ± 4.8	1.7.E−30	41.7 ± 16.7	43.4 ± 16.7	0.723
Skin type (I:II:III:IV)	5:4:3:13	16:4:4:2	–	8:7:3:7	13:1:4:8	–
Skin irritation by environment (Y/N)	14/11	5/21	–	8/17	11/15	–
Skin irritation by cosmetics (Y/N)	3/22	0/26	–	1/24	2/24	–
Cosmetic side effects (Y/N)	5/20	1/25	–	3/22	3/23	–
Use of sunscreen (“everyday”:“occasionally”:“never”)	6:8:11	12:7:7	–	3:5:17	15:10:1	–
**Moisture (AU)**						
Cheek	42.1 ± 14.0	54.3 ± 15.6	7.8.E−03	41.2 ± 12.5	55.2 ± 16.0	0.001
Forehead	60.0 ± 12.1	59.7 ± 15.5	0.836	55.4 ± 13.3	64.0 ± 13.2	0.025
**TEWL (g/m**^**2**^**h)**						
Cheek	16.8 ± 4.7	15.6 ± 7.0	0.21	19.4 ± 5.5	13.0 ± 4.5	3.7.E−05
Forehead	19.3 ± 9.2	17.2 ± 6.2	0.351	20.6 ± 5.1	16.0 ± 9.4	0.035
Sebum (μg/cm^2^)	83.4 ± 51.8	68.5 ± 46.1	0.224	98.8 ± 50.7	53.7 ± 36.0	0.001
**Skin texture (**μm**)**						
Ra	23.5 ± 5.5	23.6 ± 6.5	0.836	26.8 ± 6.2	20.4 ± 3.7	3.9.E−05
Rmax	124.8 ± 68.2	128.6 ± 74.4	0.917	141.0 ± 80.9	113.0 ± 57.8	0.160
**Periorbital wrinkle**						
Average wrinkle depth (μm)	53.2 ± 18.1	92.1 ± 25.5	1.7.E−06	77.2 ± 31.5	69.1 ± 27.4	0.332
Wrinkle volume (mm^3^)	1.7 ± 1.1	3.8 ± 1.7	9.5.E−06	3.0 ± 2.0	2.5 ± 1.5	0.309
Wrinkle area (mm^2^)	29.2 ± 10.8	40.9 ± 11.5	8.0.E−04	35.7 ± 13.6	34.7 ± 11.6	0.783
Redness (a*value)						
Cheek	10.6 ± 2.3	11.2 ± 2.4	0.274	12.0 ± 2.5	9.8 ± 1.7	0.001
Forehead	11.3 ± 1.9	11.5 ± 2.4	0.865	12.0 ± 2.4	10.8 ± 1.8	0.052
Mole (amount)	133.8 ± 45.0	212.4 ± 62.6	2.1.E−05	211.6 ± 65.6	137.6 ± 45.8	2.3.E−05
UV_spot (number)	270.1 ± 162.1	427.5 ± 162.8	1.2.E−03	263.0 ± 164.4	434.3 ± 153.2	3.4E−04
Brown_spot (number)	395.4 ± 94.3	554.3 ± 101.2	5.8.E−06	429.2 ± 116.1	521.8 ± 119.8	0.007
Porphyrin (amount)	2485.3 ± 1371.0	1675.3 ± 1465.0	0.025	2582.3 ± 1272.4	1582.0 ± 1490.2	0.013
**Skin tone (mean)**						
Forehead	194.9 ± 16.6	192.4 ± 15.4	0.427	181.4 ± 10.7	205.3 ± 7.9	1.1.E−10
Cheek	209.4 ± 7.8	204.9 ± 9.9	0.12	200.3 ± 6.4	213.6 ± 6.1	8.6.E−10

Data is expressed as the mean ± standard deviation. Skin type I:II:III:IV = dry:oily:normal:combination; AU, arbitrary unit; TEWL, transepidermal water loss; Ra, arithmetic average roughness; Rmax, maximum roughness depth.

### Alpha diversity and beta diversity

**Figure 1:**
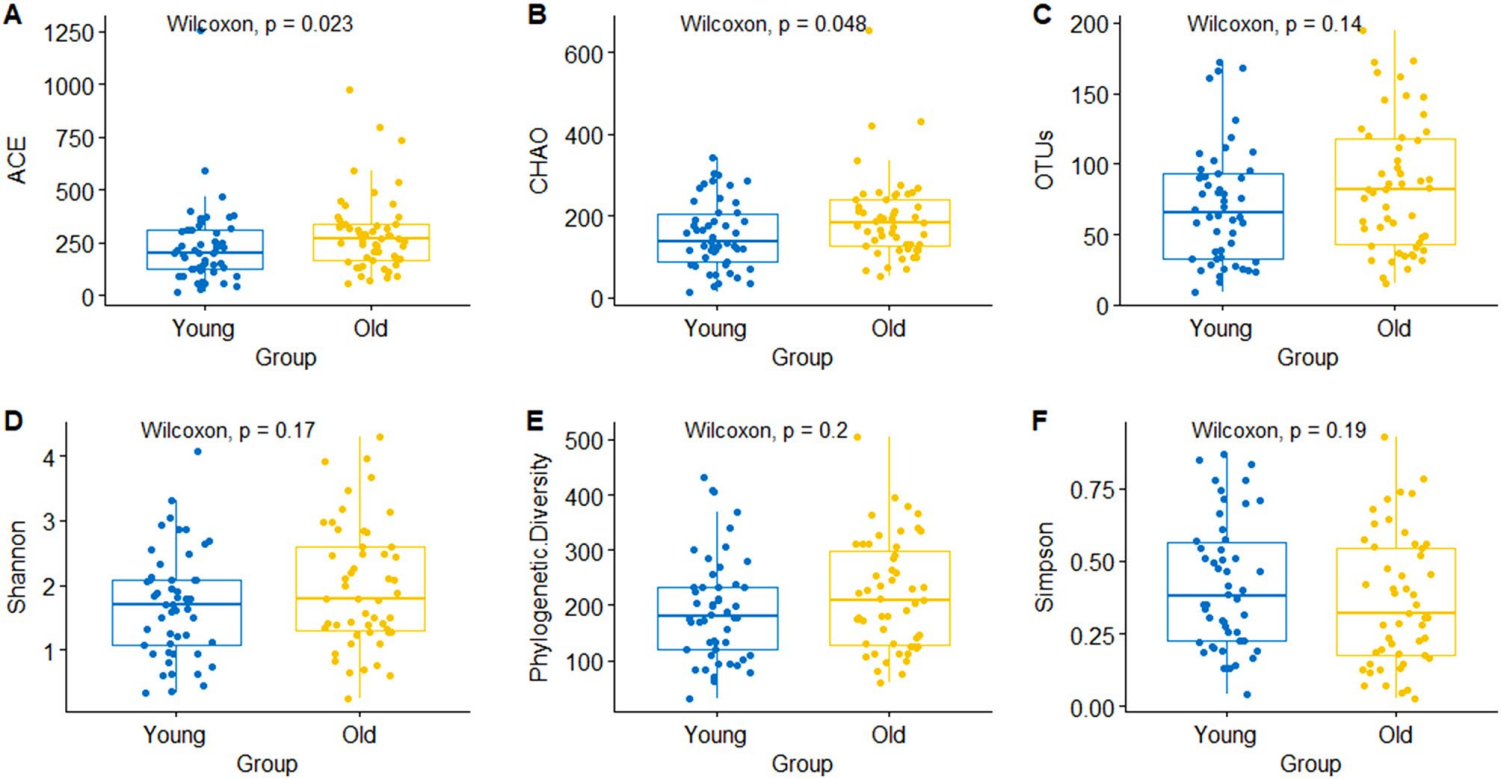
Alpha-diversity comparison between the younger (young) and older (old) groups. (**A**) Abundance-based coverage estimator (ACE); (**B**) Chao1-estimated OTU number; (**C**) OTUs; (**D**) Shannon's diversity; (**E**) phylogenetic diversity; (F) Simpson's Diversity Index. Wilcoxon signed-rank test was used for statistical analysis. *P* values are displayed in the figures.

The bacterial 16S rRNA gene V3–V4 region in the skin collection sample was sequenced using the Illumina Miseq platform, and the total number of reads confirmed after quality check for the raw sequence was 5,514,434. The average number of sequencing reads for 102 samples was 55,144, and the average number of reads for each group was 56,089.98 (younger group) and 54,198.7 (older group), respectively. A total of 36,344 OTUs were identified using the UCLUST tool, clustering the 16S rRNA sequences with a ≥ 97% identity threshold for taxonomic profiling analysis. The total number of OTUs in the younger group was 16,963 and the total number in the older group was 19,381. Before analyzing the difference between the two age groups, all reads were normalized to reduce the bias due to the number of samples. We compared the species richness, alpha-diversity indices such as Abundance-based Coverage Estimator (ACE) and Chao1-estimated OTU number between the two groups classified by age (Fig. 1A and B). There was no significant difference in OTU counts between the younger and older groups (Fig. 1C). The Wilcoxon rank-sum test evaluated the two species richness indices. The ACE index was significantly higher in the older group than the younger group; (*P* = 0.023). In addition, the older group had significantly higher Chao1-estimated OTUs (*P* = 0.048). The species diversity indices, Shannon's diversity, Phylogenetic diversity, and Simpson's diversity index showed no significant differences (Fig. 1D–F).

**Figure 2:**
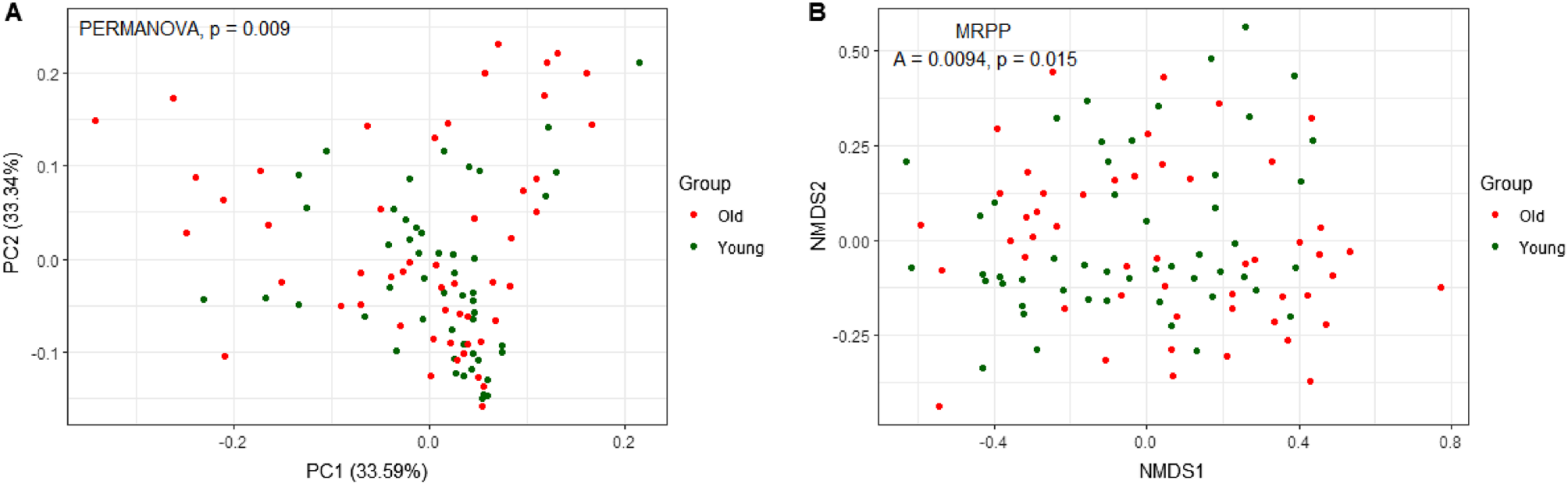
Beta diversity between the younger (young) and older (old) groups. (**A**) Principal coordinates analysis (PCoA) plot of UniFrac distances; (**B**) non-metric multidimensional scaling (NMDS) plot of generalized UniFrac distances. Red circles represent the older group, and green circles represent the younger group.* P* values are displayed in the figures.

The analysis of beta diversity was executed at the species level of phylogenetic results to confirm the difference in distance between the two age groups. The UniFrac Principal Coordinates Analysis (PCoA) showed a significant difference between the two age groups (Fig. 2A, P = 0.009). The non-metric multidimensional scaling (NMDS) plot based on the Bray–Curtis dissimilarity also showed a significant difference. However, the delta variance was similar between the two age groups; that is, the difference within groups was not significant (Chance corrected within-group agreement A = 0.0094, *P* = 0.015).

In addition, we investigated the difference in alpha and beta diversities according to gender, but we could not find any significant difference (Supplementary Fig. 1).

### Differences in relative abundance

**Figure 3:**
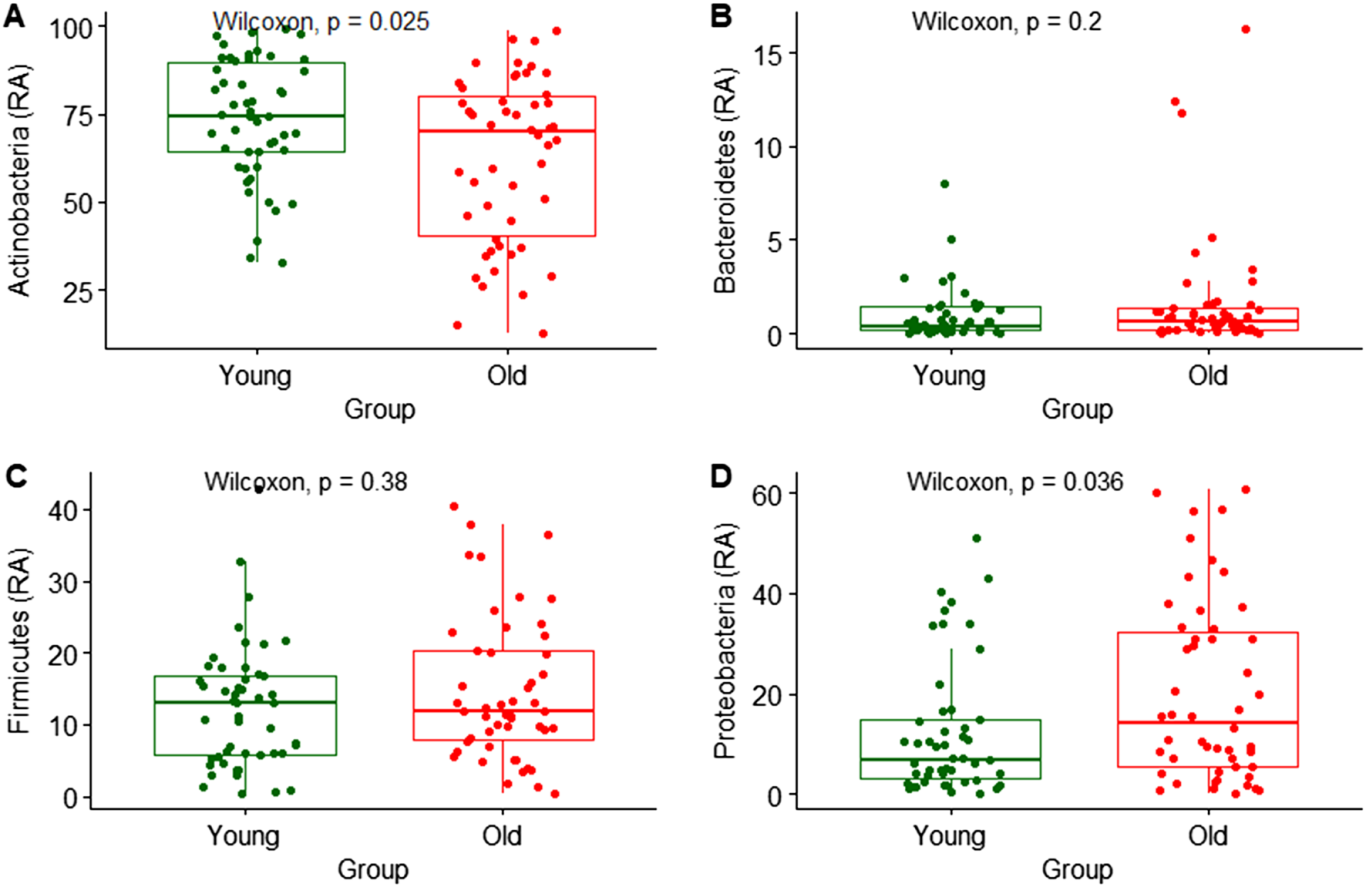
Relative abundance (%) of four dominant phyla in skin samples between younger (young) and older (old) group. (**A**) Actinobacteria; (**B**) Bacteroidetes; (**C**) Firmicutes; (**D**) Proteobacteria. Wilcoxon signed-rank test was used for statistical analysis. *P* values are displayed in the figures.

**Figure 4:**
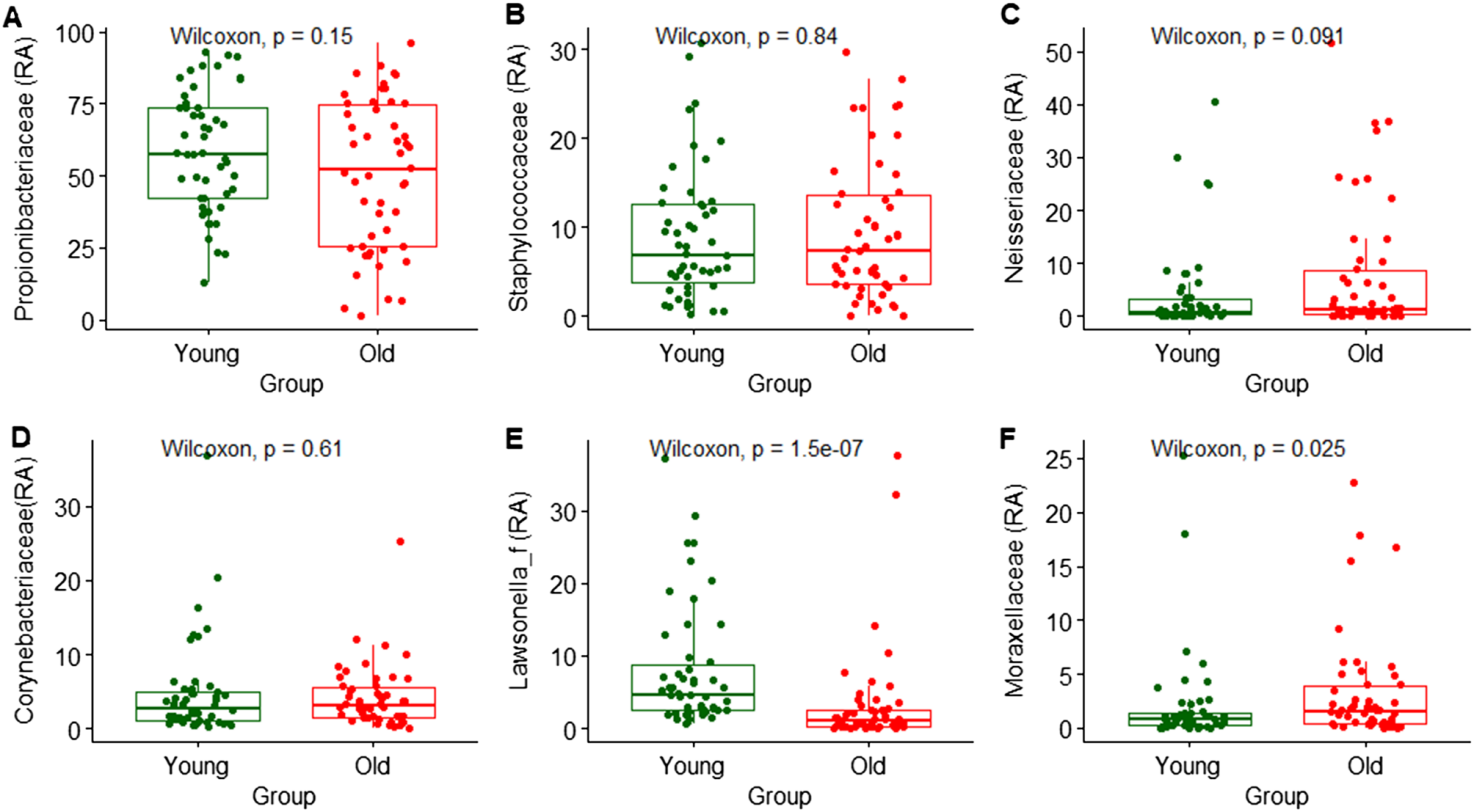
Relative abundance (%) of the six dominant families in skin samples between younger (young) and older (old) group. (**A**) Propionibacteriaceae; (**B**) Staphylococcaceae; (**C**) Neisseriaceae; (**D**) Corynebacteriaceae; (**E**) Lawsonella; (**F**) Morganellaceae. Wilcoxon signed-rank test was used for statistical analysis. *P* values are displayed in the figures.

First, we focused on differences in the relative abundance at the phylum level between the two age groups. Figure 3 indicates the distribution of the four dominant phyla: Actinobacteria, Bacteroidetes, Firmicutes, and Proteobacteria. The most predominant taxonomic group at the phylum level between the two age groups was Actinobacteria and the average abundances were 62.3% and 73.4% in the younger and older groups, respectively (*P* = 0.025). Supplementary Figs. 2 and 3 indicate the analysis results for all skin microbial compositions. Regarding the distribution of Proteobacteria, which is also known to dominate human skin bacteria, the average abundance was significantly higher in the older group than Actinobacteria. At the family level analysis of skin samples, Fig. 4 indicates the distribution of the six most abundant for younger and older groups. The compositions of *Lawsonella* and *Morganellaceae* were significantly different according to age groups (*P* < 0.05).

**Figure 5:**
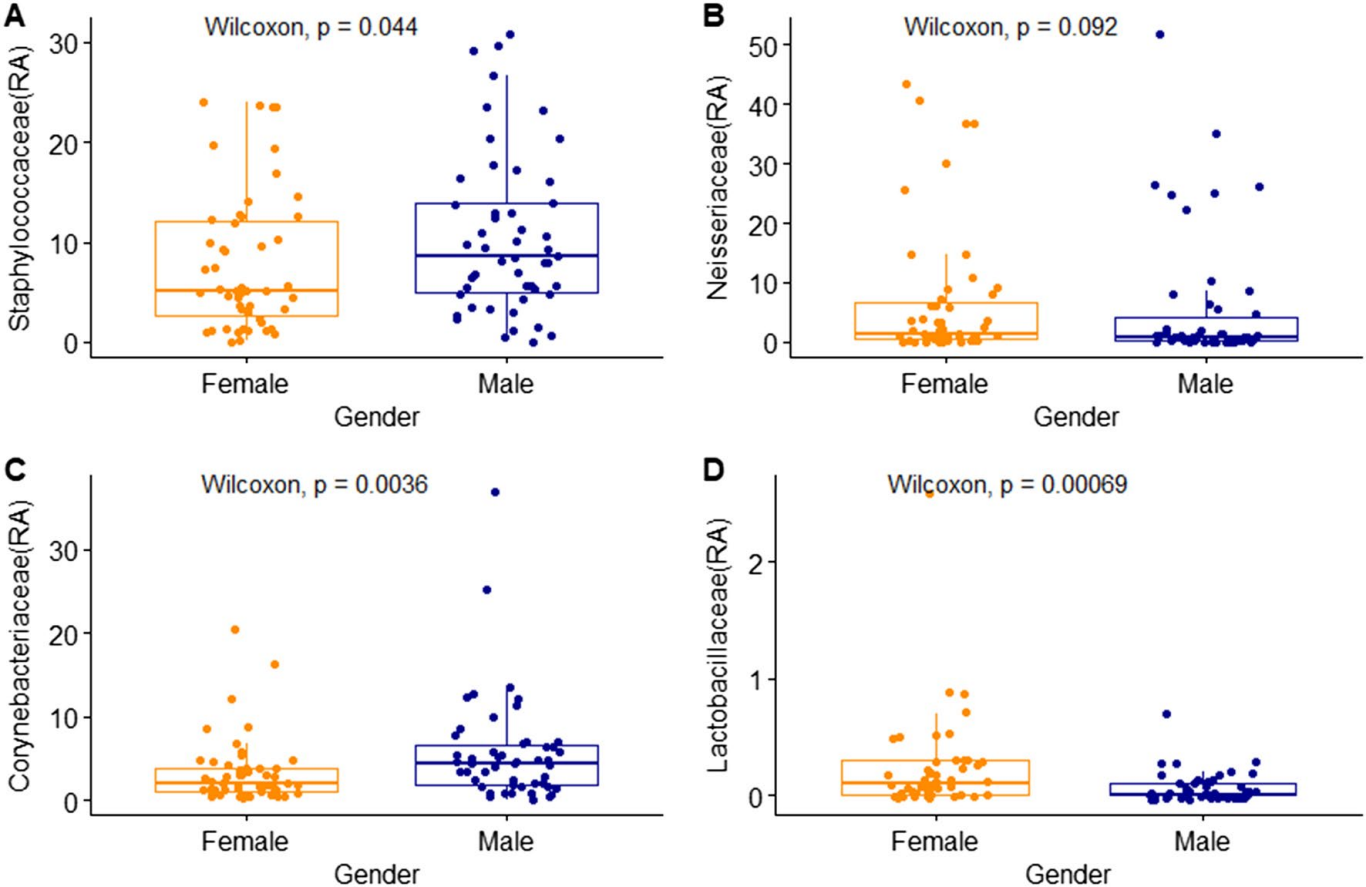
Relative abundance (%) of four families in skin samples between female and male. (**A**) Staphylococcaceae; (**B**) Neisseriaceae; (**C**) Corynebacteriaceae; (**D**) Lactobacillaceae. Wilcoxon signed-rank test was used for statistical analysis. *P* values are displayed in the figures.

**Figure 6:**
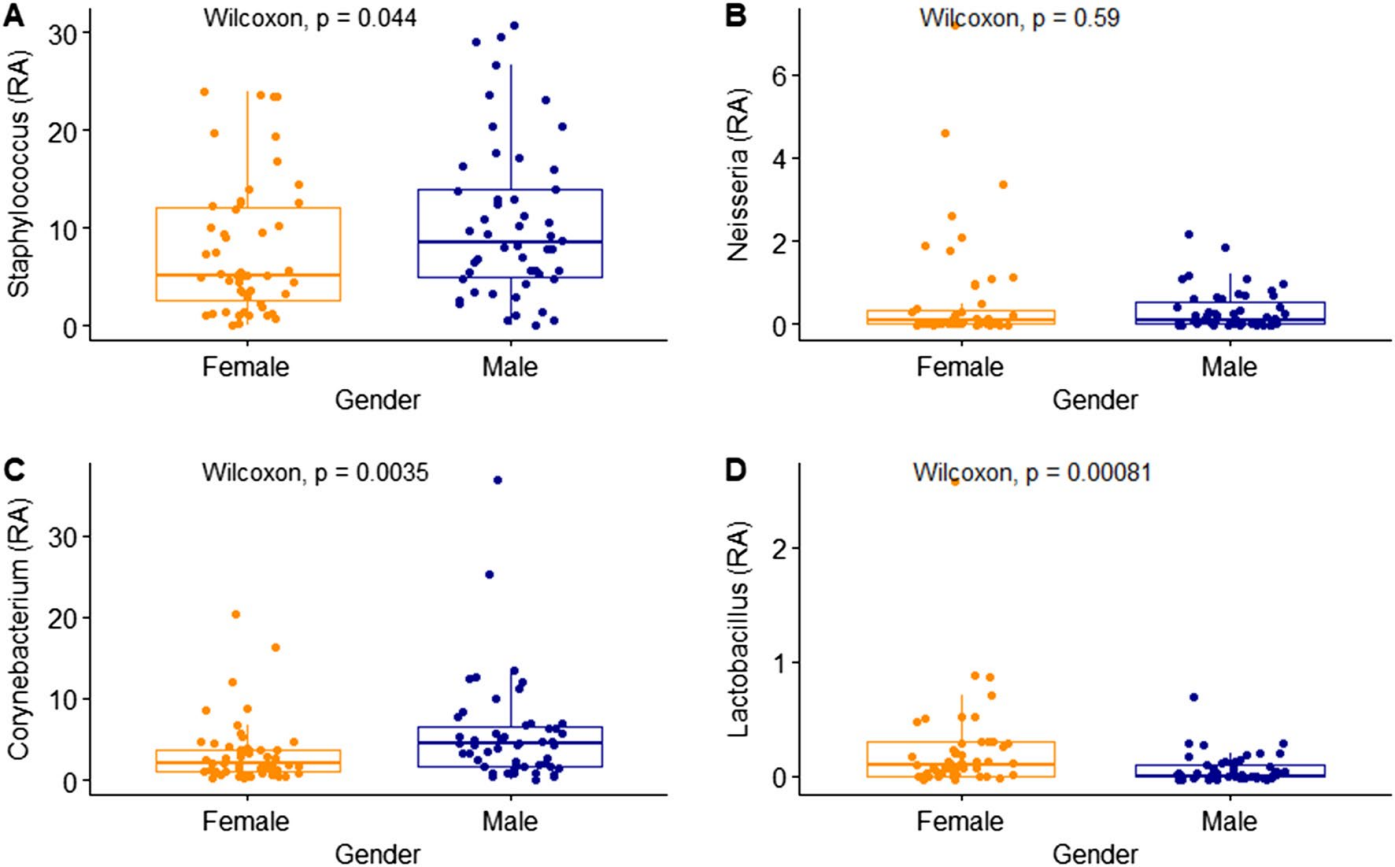
Relative abundance (%) of four genera in skin samples between female and male. (**A**) *Staphylococcus*; (**B**) *Neisseria*; (**C**) *Corynebacterium*; (**D**) *Lactobacillus*. Wilcoxon signed-rank test was used for statistical analysis. *P* values are displayed in the figures.

In addition, differences in the distribution of skin microorganisms according to gender were confirmed at the phylum level, family level, and species level. There were no significant differences in the four major skin phyla (Supplementary Fig. 4). Figure 5 indicates the relative abundance of the four dominant families between the male and female groups. Staphylococcaceae and Corynebacteriaceae showed a high distribution in the male group; Lactobacillaceae, which belongs to the lactic acid bacteria family, showed a higher relative abundance in the female group. The difference in distribution at the family level was similar to the genus level; *Staphylococcus* and *Corynebacterium* were significantly higher in the male group and *Lactobacillus* was significantly higher in the female group (Fig. 6).

**Figure 7:**
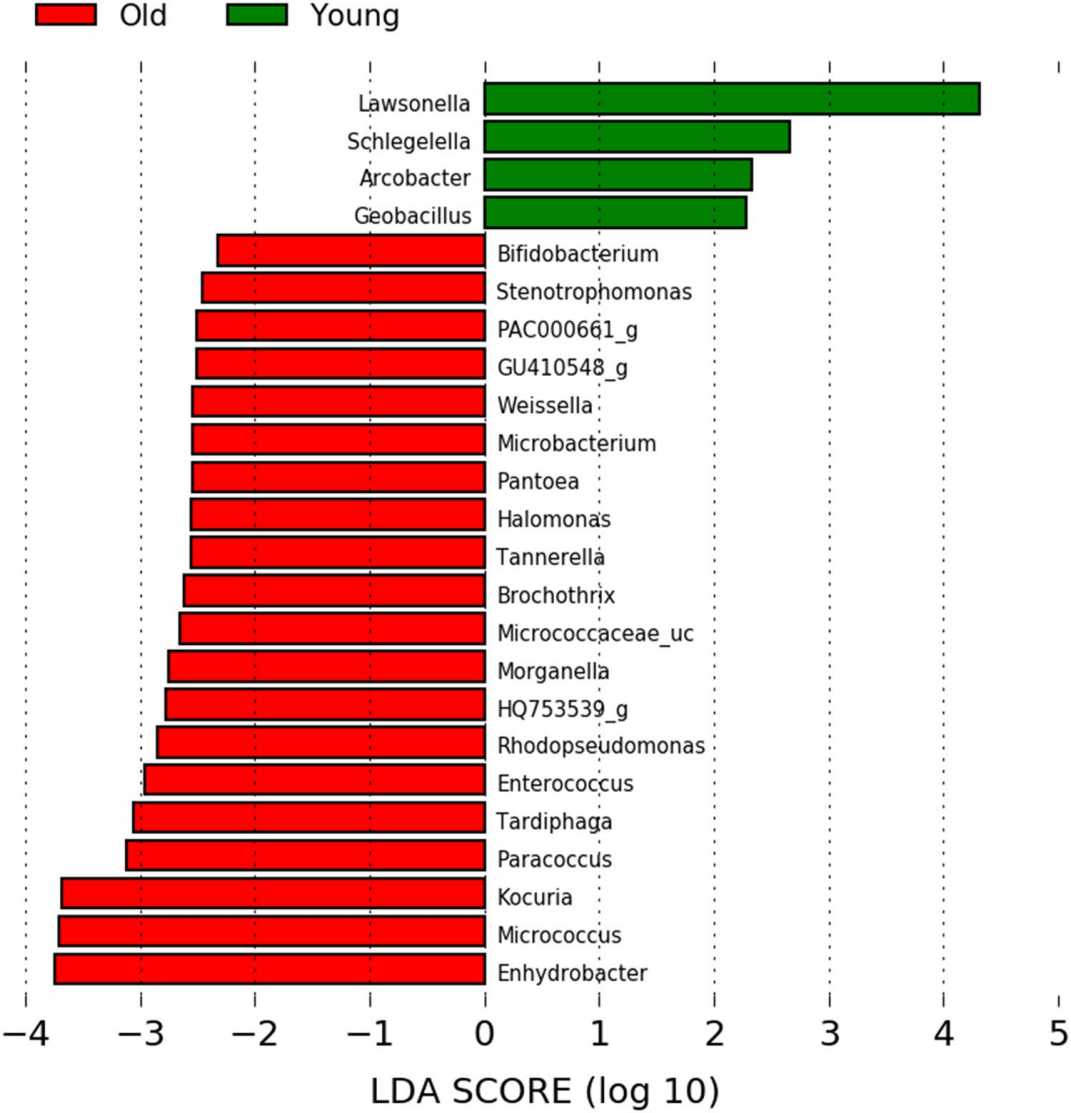
Taxonomic differences from LEfSe analysis. Linear discriminative analysis (LDA) scores are calculated at the genus level between the younger group (green, young) and the older group (red, old).

**Figure 8:**
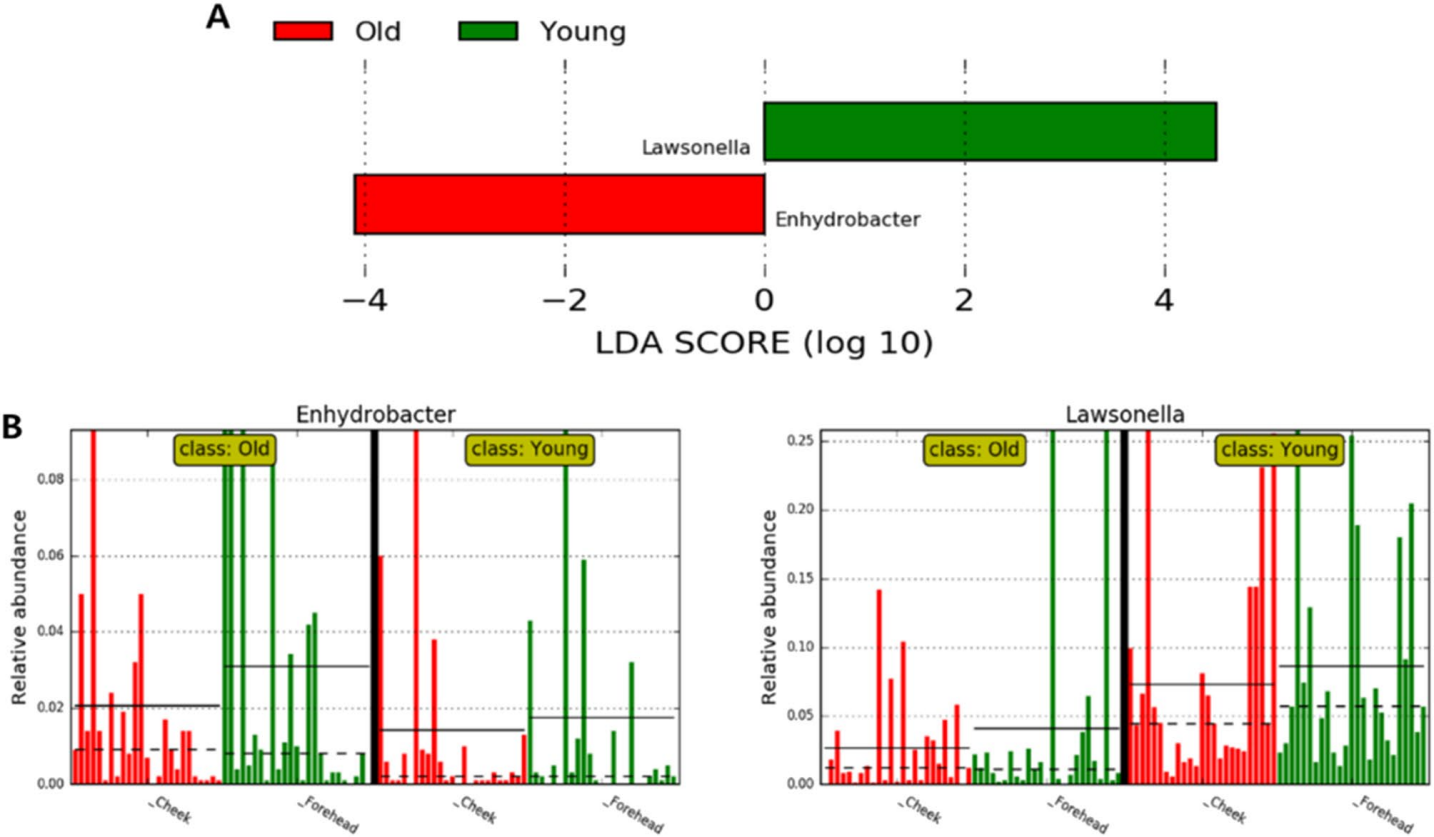
Taxonomic differences from LEfSe analysis. (**A**) Linear discriminative analysis (LDA) scores are calculated at the genus level with subcategories of skin site between younger group (green) and older group (red); (**B**) Relative abundance (%) of dominant genera in skin site (cheek and forehead) classified by age.

The linear discriminant analysis (LDA) model identifies differently abundant taxa between groups and estimates each significantly different taxon's effect size^22,23^. We evaluated the LDA effect size (LEfSe) among groups to search for a statistically significant biomarker at the genus level. The LEfSe of all genera showed 24 bacterial taxa with significant differences (Fig. 7). LEfSe analysis revealed that the genus *Lawsonella* was the most abundant in the younger group, and *Enhydrobacter* was the most abundant in the older group. Additionally, we analyzed LEfSe by including categories for each skin site (cheek and forehead) as a subclass. It was confirmed between the two groups that *Lawsonella* and *Enhydrobacter* showed significant differences (|LDA score|> 4.0) as the results of LEfSe analysis of genera classified by age (Fig. 8A). Figure 8B indicates the relative abundances of the dominant genus in the skin site classified by age.

LDA was conducted according to gender; Table 2 indicates the LEfSe of all taxonomic biomarker (taxon) without < 0.1% abundance (|LDA score|> 2.5). Likewise, our previous analysis results of the distribution of skin microbiota by gender, Corynebacteriaceae, was widely distributed in males and the LDA effect size was the largest at the family level. At the genus level, the LDA effect size of *Corynebacterium* and *Staphylococcu*s was also larger in the male group. Notably, the largest LDA effect size was the distribution of the genus *Xanthomonas* between the male and female groups. LDA analysis confirmed that *Xanthomonas citri* were present at a higher proportion in the female group and *Corynebacterium tuberculostearicum* strains were present in a higher proportion in the male group. Moreover, *Enterococcus faecalis*, which is a type of lactic acid bacteria, was widely distributed in women and the LDA effect size of *Lactobacillus helveticus* was 2.34, which had a higher distribution in the female group (Table 2 and Supplementary Fig. 5).

**Table 2:** Skin characteristics of the subjects by gender.

Taxon name	Taxon rank	LDA effect size	*P* value	*P* value (FDR)	Female	Male
Saccharibacteria_TM7	Phylum	2.63569	0.01880	0.44544	0.11570	0.03000
Oligoflexia	Class	3.54592	0.02763	0.44544	0.98630	0.29200
Saccharimonas_c	Class	2.62516	0.01921	0.44544	0.11370	0.03000
Bacillales	Order	4.10532	0.04581	0.44544	7.94710	10.77800
Rhizobiales	Order	3.71175	0.03682	0.44544	1.61370	0.52200
Rhodobacterales	Order	3.67928	0.02134	0.44544	0.33140	1.22000
Bdellovibrionales	Order	3.55566	0.02904	0.44544	0.98240	0.29000
Saccharimonas_o	Order	2.61017	0.01921	0.44544	0.11370	0.03000
Corynebacteriaceae	Family	4.12008	0.00360	0.44544	3.31760	5.71200
Staphylococcaceae	Family	4.10209	0.04365	0.44544	7.77250	10.59400
Rhodobacteraceae	Family	3.68614	0.02709	0.44544	0.32940	1.21800
Bradyrhizobiaceae	Family	3.51290	0.00042	0.42351	0.85100	0.17600
Pseudomonadaceae	Family	3.12226	0.02418	0.44544	0.53920	0.25200
Lactobacillaceae	Family	2.93605	0.00068	0.42351	0.22750	0.06600
Nocardiaceae	Family	2.82280	0.03940	0.44544	0.30980	0.20400
Saccharimonas_f	Family	2.61652	0.00445	0.44544	0.10200	0.01800
Mycobacteriaceae	Family	2.60005	0.02135	0.44544	0.11960	0.03600
*Xanthomonas*	Genus	4.13951	0.00170	0.44544	2.60980	0.03200
*Corynebacterium*	Genus	4.12004	0.00344	0.44544	3.31570	5.71000
*Staphylococcus*	Genus	4.10149	0.04329	0.44544	7.76860	10.58600
*Paracoccus*	Genus	3.68396	0.03572	0.44544	0.23920	1.16200
*Bradyrhizobium*	Genus	3.23179	0.00099	0.43426	0.50390	0.17400
*Acinetobacter*	Genus	3.21231	0.01345	0.44544	0.69610	0.35000
*Elizabethkingia*	Genus	3.17255	0.04441	0.44544	0.35880	0.00000
*Pseudomonas*	Genus	3.10444	0.02418	0.44544	0.53920	0.25200
*Tardiphaga*	Genus	2.97900	0.04441	0.44544	0.23730	0.00000
*Lactobacillus*	Genus	2.91675	0.00080	0.42351	0.22550	0.06600
*Mycobacterium*	Genus	2.60728	0.02135	0.44544	0.11960	0.03600
*Rhodopseudomonas*	Genus	2.59941	0.04441	0.44544	0.09610	0.00000
*Saccharimonas*	Genus	2.52382	0.00789	0.44544	0.08430	0.01600
*Xanthomonas citri* group	Species	4.15332	0.00170	0.44544	2.60780	0.03200
*Corynebacterium tuberculostearicum*	Species	4.05179	0.00000	0.01166	1.43920	3.67000
*Paracoccus denitrificans* group	Species	3.69971	0.01347	0.44544	0.16080	1.09400
*Anaerococcus nagyae* group	Species	3.60895	0.00463	0.44544	0.05490	1.03800
*Bradyrhizobium japonicum* group	Species	3.25236	0.00114	0.43844	0.50200	0.17400
*Elizabethkingia miricola* group	Species	3.17206	0.04441	0.44544	0.35690	0.00000
*Cutibacterium granulosum*	Species	3.10884	0.02549	0.44544	0.23330	0.55000
*Tardiphaga robiniae*	Species	2.95101	0.04441	0.44544	0.22940	0.00000
*Acinetobacter proteolyticus* group	Species	2.72316	0.01247	0.44544	0.18630	0.08200
*Corynebacterium xerosis* group	Species	2.70282	0.04465	0.44544	0.12550	0.01000
*Streptococcus sanguinis* group	Species	2.67887	0.00339	0.44544	0.10000	0.17400
*Enterococcus faecalis*	Species	2.63833	0.01658	0.44544	0.12160	0.04200
*Corynebacterium minutissimum* group	Species	2.59029	0.01809	0.44544	0.03330	0.10800
*Pseudomonas amygdali* group	Species	2.52548	0.00825	0.44544	0.08430	0.00200

Values in the table were presented through linear discriminant analysis (LDA) effect size (LEfSe). *FDR* false discovery rate; Female, an average of relative abundance in the female group; Male, an average of relative abundance in the male group.

### Correlation between skin symbiotic bacteria and clinical skin parameters

**Figure 9:**
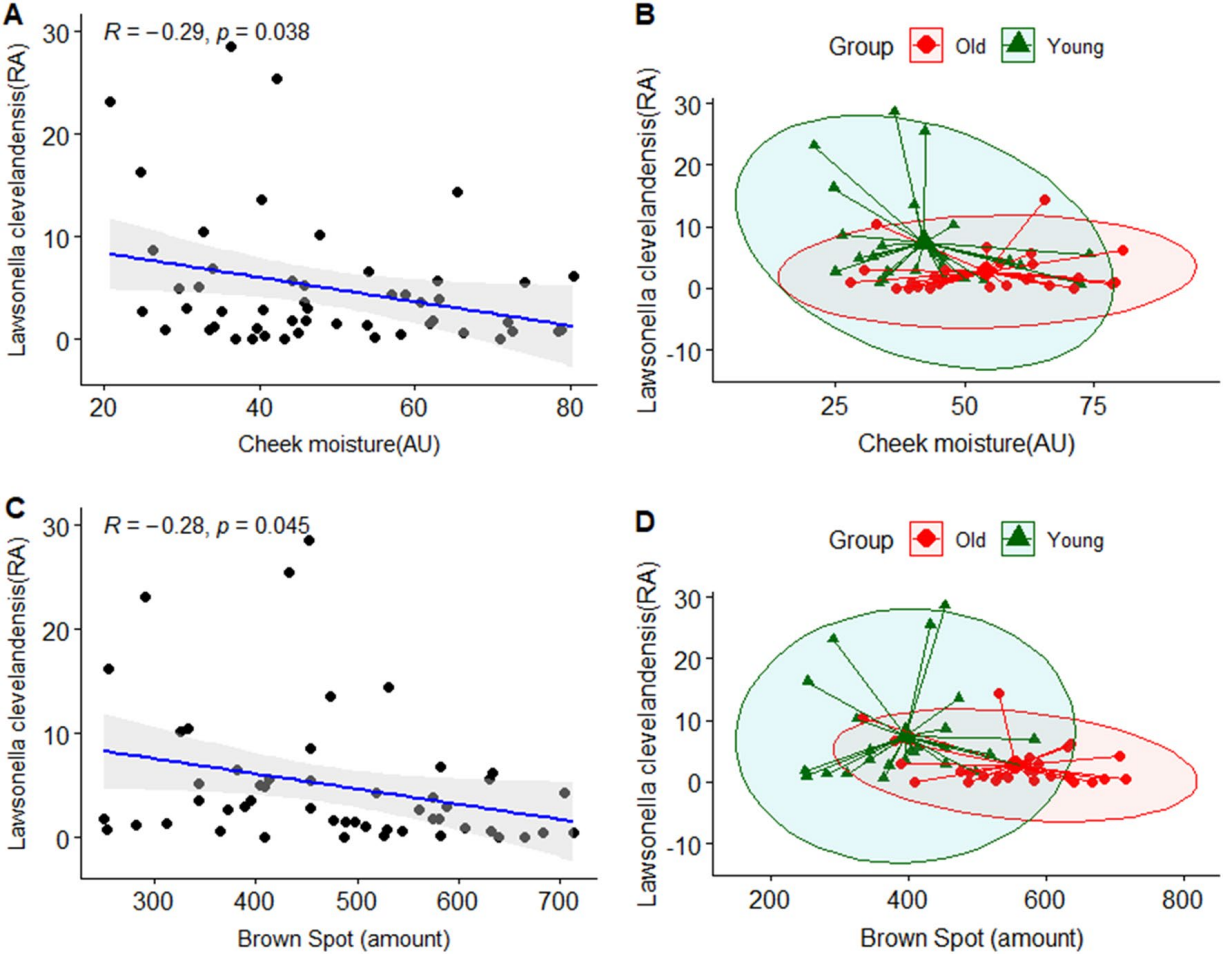
Correlation analysis between the relative abundance (%) of *Lawsonella clevelandensis* and clinical skin parameters. (**A**) Pearson correlation between the relative abundance (%) of *Lawsonella clevelandensis* and cheek moisture (AU); (**B**) age-related Pearson correlation between the relative abundance (%) of *Lawsonella clevelandensis* and cheek moisture (AU); (**C**) Pearson correlation between the relative abundance (%) of *Lawsonella clevelandensis* and brown spots (amount); (**D**) age-related Pearson correlation between the relative abundance (%) of *Lawsonella clevelandensis* and brown spots (amount). The green color indicates the younger group, and the red color indicates the older group.

**Figure 10:**
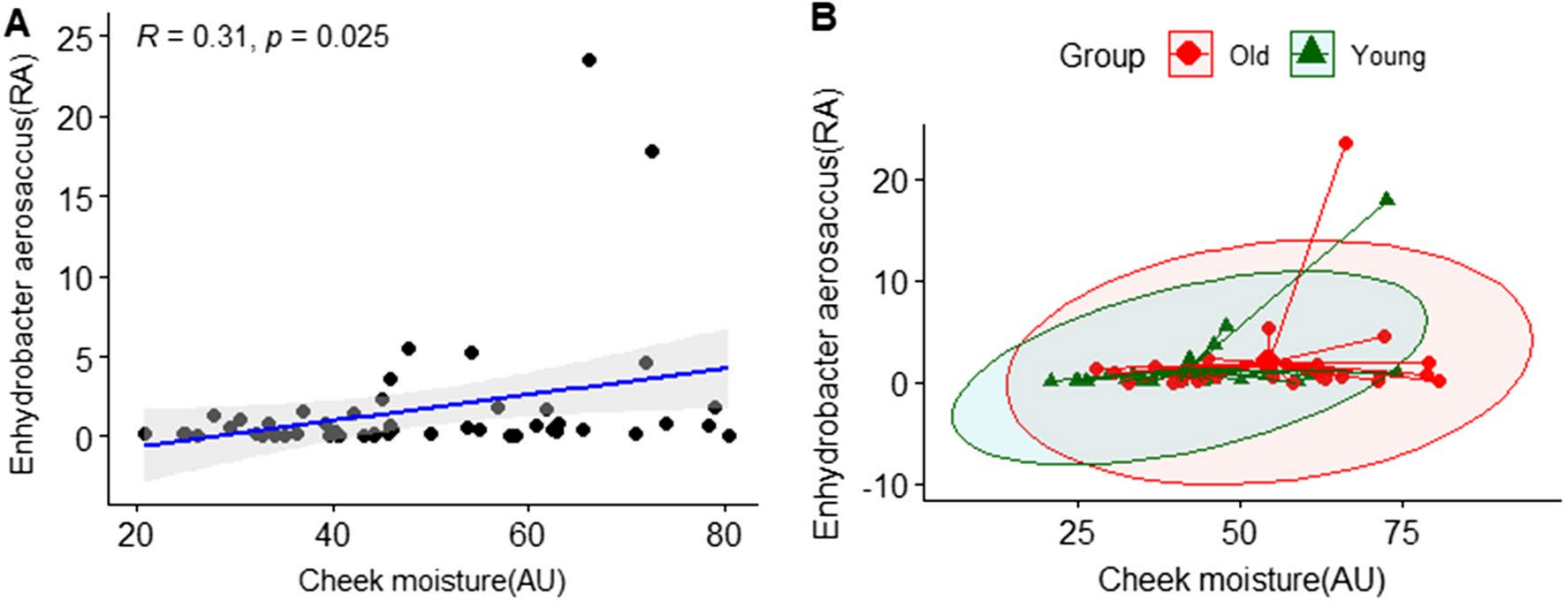
Correlation analysis between the relative abundance (%) of *Enhydrobacter aerosaccus* and clinical skin parameters. (**A**) Pearson correlation between the relative abundance (%) of *Enhydrobacter aerosaccus* and cheek moisture (AU); (**B**) age-related Pearson correlation between the relative abundance (%) of *Enhydrobacter aerosaccus* and cheek moisture (AU). The green color indicates the younger group, and the red color indicates the older group.

**Figure 11:**
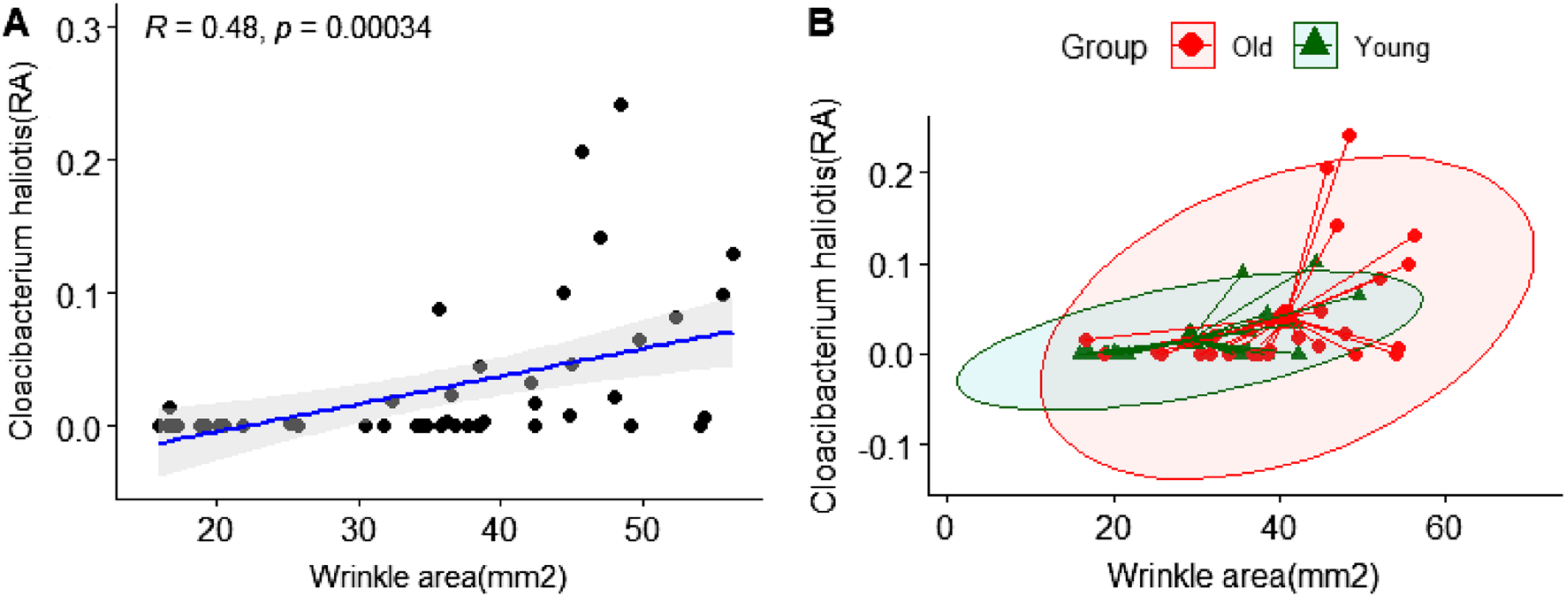
Correlation analysis between the relative abundance (%) of *Cloacibacterium haliotis* and clinical skin parameters. (**A**) Pearson correlation between the relative abundance (%) of *Cloacibacterium haliotis* and periorbital wrinkle area (mm^2^); (**B**) age-related Pearson correlation between the relative abundance (%) of *Cloacibacterium haliotis* and periorbital wrinkle area (mm^2^). The green color indicates the younger group, and the red color indicates the older group.

Based on the results of LEfSe analysis, we investigated which skin clinical parameters correlated with the relative abundance of skin microflora. We analyzed Pearson correlation, which was classified by skin site (cheek and forehead) between the bacterial taxa at a species level and the subjects’ clinical skin parameters. The cheek skin parameters, moisture content, and amount of brown spots were negatively correlated with the *Lawsonella clevelandensis* group belonging to the genus *Lawsonella* of subjects with statistical significance. (Fig. 9). The skin parameter-related correlation of *Lawsonella clevelandensis* in the younger group showed that the gradient between the cheek moisture content and abundance of *Lawsonella clevelandensis* was more negatively inclined. Moreover, regarding the gradient between the amount of brown spots and the abundance of *Lawsonella clevelandensis,* both groups showed similar patterns (Figs. 9B and 9D). The *Enhydrobacter aerosaccus* group belonging to the genus *Enhydrobacter* was positively correlated with the cheek moisture content with statistical significance. (Fig. 10). However, there was no significant correlation with the forehead skin parameters of either of the two species. Figure 11 shows the skin parameter-related correlation with the relative abundance of *Cloacibacterium haliotis*. There was a high correlation coefficient between *the* abundance of *Cloacibacterium haliotis* and the periorbital wrinkle area (R = 0.48).

**Figure 12:**
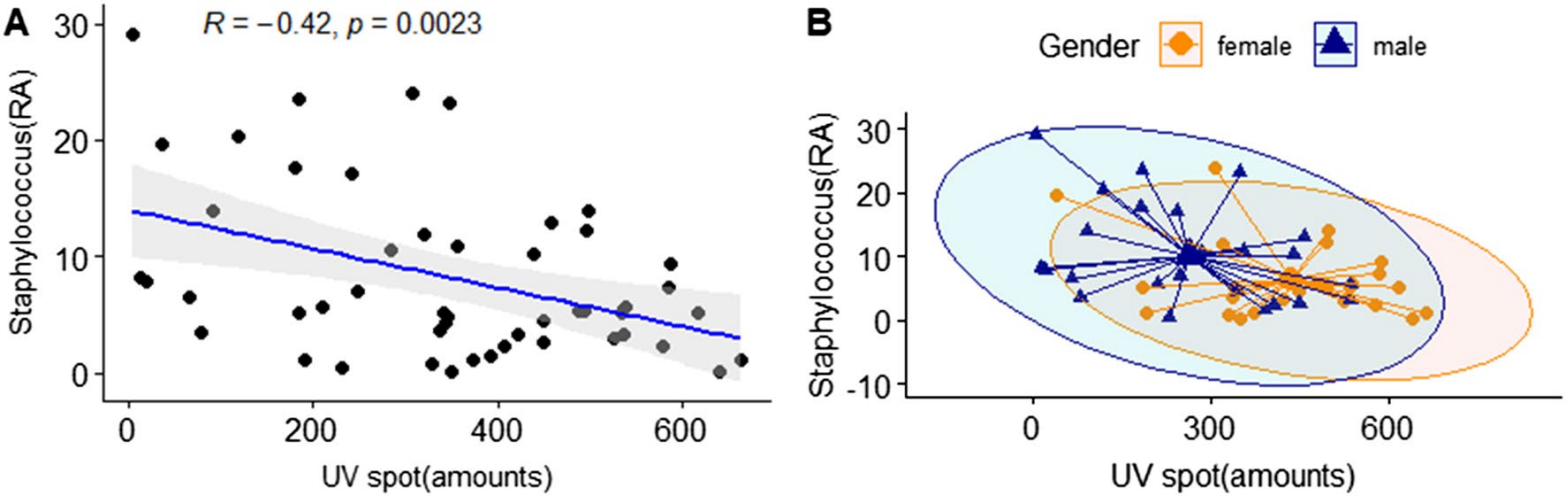
Correlation analysis between the relative abundance (%) of *Staphylococcus* and clinical skin parameters. (**A**) Pearson correlation between the relative abundance (%) of *Staphylococcus* and UV spots (amounts); (**B**) gender-related Pearson correlation between the relative abundance (%) of *Staphylococcus* and UV spots (amounts).

**Figure 13:**
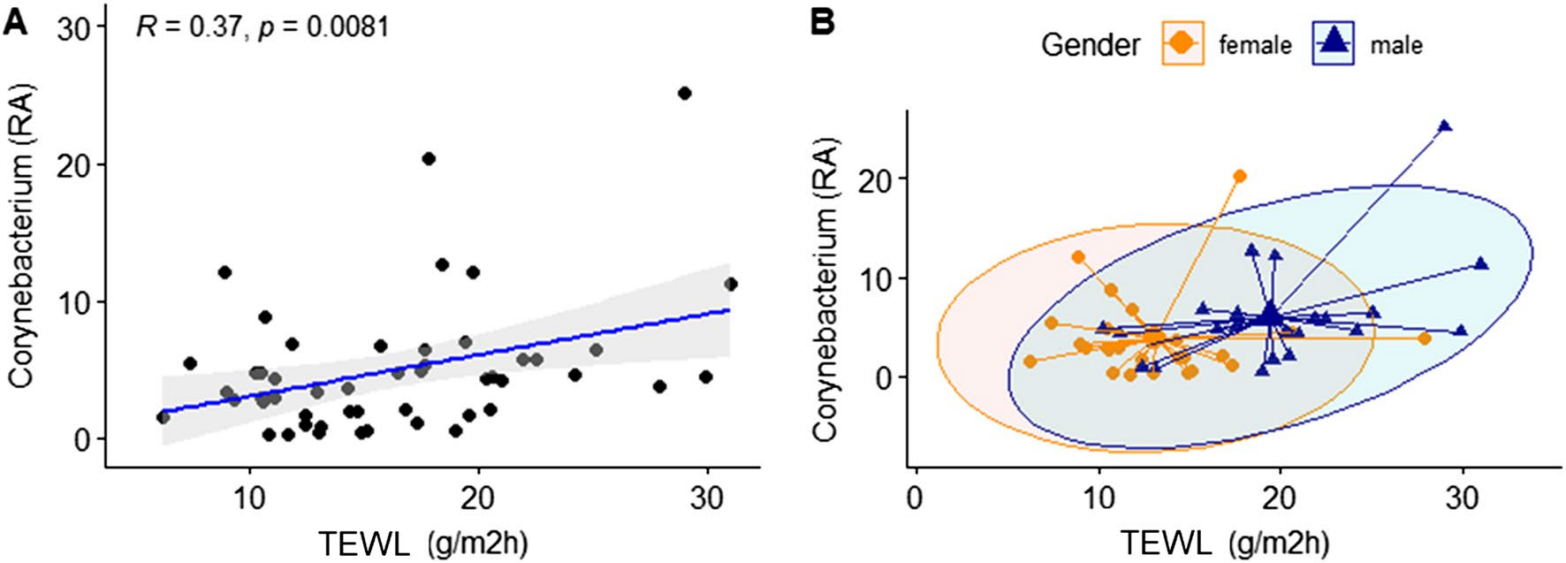
Correlation analysis between the relative abundance (%) of *Corynebacterium* and clinical skin parameters. (**A**) Pearson correlation between the relative abundance (%) of *Corynebacterium* and transepidermal water loss (g/m^2^h); (**B**) gender-related Pearson correlation between the relative abundance (%) of *Corynebacterium* and transepidermal water loss (g/m^2^h).

**Figure 14:**
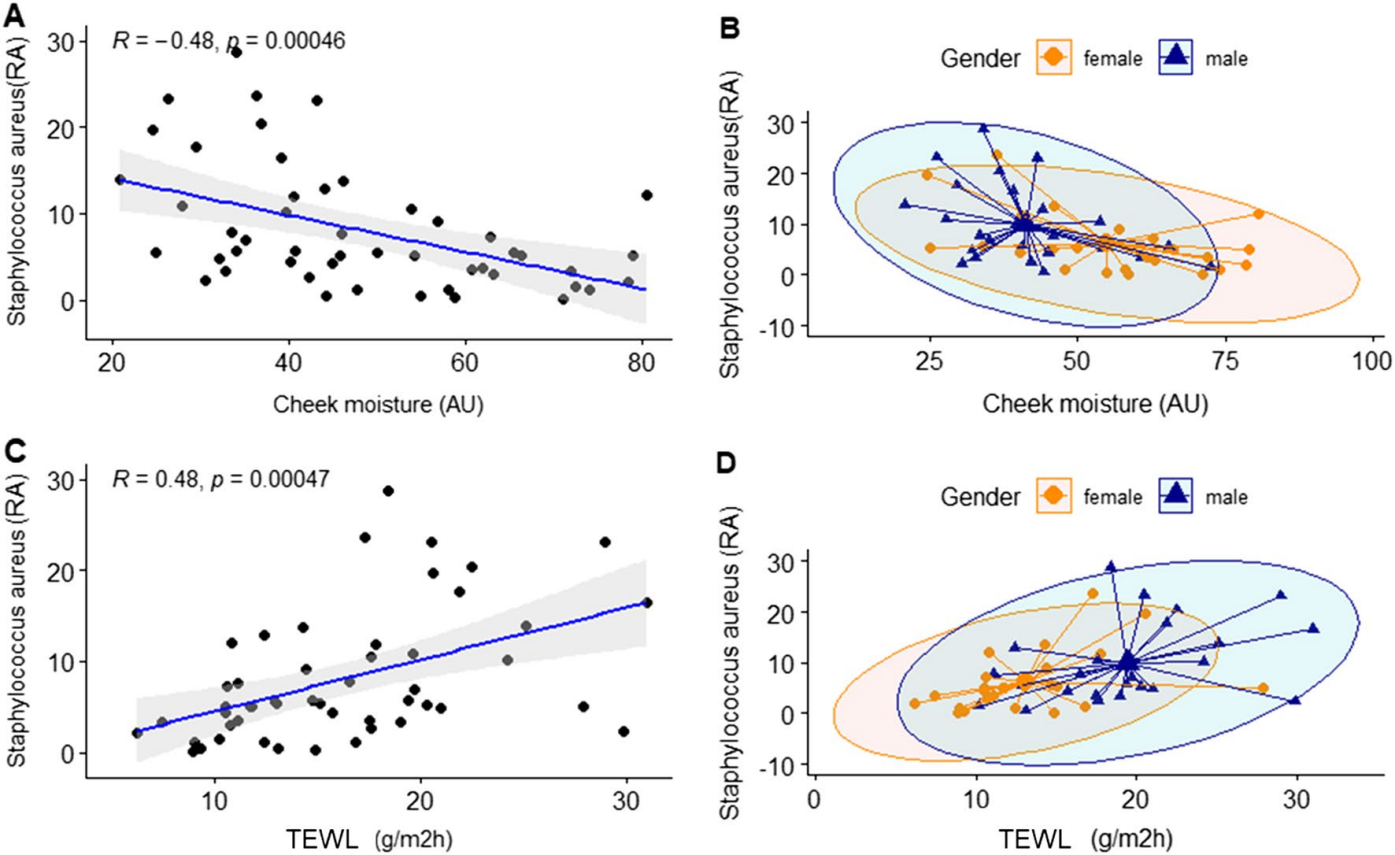
Correlation analysis between the relative abundance (%) of *Staphylococcus aureus* and clinical skin parameters. (**A**) Pearson correlation between the relative abundance (%) of *Staphylococcus aureus* and cheek moisture (AU); (**B**) gender-related Pearson correlation between the relative abundance (%) of *Staphylococcus aureus* and cheek moisture (AU); (**C**) Pearson correlation between the relative abundance (%) of *Staphylococcus aureus* and transepidermal water loss (g/m^2^h); (**D**) gender-related Pearson correlation between the relative abundance (%) of *Staphylococcus aureus* and transepidermal water loss (g/m^2^h).

We analyzed the correlation between gender and clinical skin parameters for the genus that showed significant differences. The genera *Staphylococcus* and *Corynebacterium*, which were highly dominant in male skin, were examined for their correlation with skin characteristics on subjects' cheeks and foreheads. *Staphylococcus* was negatively correlated with the number of UV spots in cheeks with statistical significance (R =  − 0.42, *P* = 0.0023). These results suggest that the reduction of the UV spots could be predicted by *Staphylococcus* abundance (Fig. 12). *Corynebacterium* was positively correlated with the TEWL in cheeks with statistical significance (Fig. 13). Moreover, we focused on the correlation between skin characteristics and the relative abundance of species of *Staphylococcus aureus*, which is known to be closely related to atopic dermatitis^24^. It was confirmed that the higher the distribution of *S. aureus*, the lower the skin moisture content in the cheeks and the higher the transdermal moisture loss (Fig. 14).

## Discussion

This study aimed to profile the distribution of facial skin microbiota according to age and gender in healthy Koreans and to confirm their association with skin characteristics. There was a significant difference in periorbital wrinkles and number of facial spots when comparing the clinical skin parameters between the younger and older age groups. These results are similar to the previous reports that reveal that chronological increase in age was related to the clinical appearance of facial wrinkles and facial hyperpigmentation^25,26^. However, the cheek moisture content was significantly higher among the elderly. This was a contrasting result with some previous reports that skin hydration decreases with age^27^. However, from other observations, there was no association between skin hydration and age^26,28^. There was no significant difference between the age groups in terms of forehead skin hydration in this study. The differences in skin characteristics according to gender were found to be more significant than the differences by age without periorbital wrinkles. In particular, the male group had higher sebum contents than the female group, which could be associated with the relative abundances of *Staphylococcus* and *Corynebacterium* that use facial sebum as nutrients^29,30^. The overall skin characteristics results confirm that the female skin had a more positive result from a cosmetic perspective but the number of UV spots was found to be higher in the female group.

In the results of skin microbiome analysis, the alpha-diversity index was significantly higher in the elderly, which was similar to previous reports that the older group showed a tendency toward a higher alpha diversity than the younger group for all skin microbiomes in Japanese women^5^. Nevertheless, there was no difference in alpha diversity by gender (data not shown). A study investigating the interactions between the host and the skin microbiome reported that the contribution of gender to skin microbial diversity likely arises as a downstream effect of male and female steroid production^31^. Since we had no gender restrictions, in contrast to the results of age-related beta diversity of Japanese women^5^ and Chinese women^10^, the distance differences between the two age groups were significant, but it was difficult to find a largely distinguished difference.

The relative abundance of skin microbiota between the younger and older groups confirmed that Actinobacteria and Proteobacteria, the two known major human skin phyla, are dominant in the younger age group and older age group, respectively. Indeed, the genus of *Lawsonella* was more predominant and *Lawsonella clevelandensis* was identified as a major species in the younger group. Recent studies showed that *L. clevelandensis* was among the most common species on the human skin, scalp, and nostrils^32–36^. Additionally, there was a significant negative correlation between *L. clevelandensis* and the cheek moisture contents and brown spots on the face, although the correlation was not high. *Enhydrobacter aerosaccus*, which has a high relative abundance in the elderly's function, has been discovered through a recently published skin microbiome study in China^37^. In that study, *E. aerosaccus* and *M. osloensis* were taxonomically considered to be the same species. Furthermore, the *M. osloensis* group is dominant in less-hydrated skin, which contrasts with the results of this study. These results can be explained by differences in the participant selection criteria, their living environment and ethnicity.

We confirmed that the genus *Staphylococcus* was significantly associated with UV spot number on cheeks and *Corynebacteria* were significantly associated with TEWL. The genera *Staphylococcus* and *Corynebacterium* dominated in males^38^, known as major normal skin bacteria, and are reportedly related to the sebum or hydration levels of the facial skin^39^. Subsequently, the distribution of *Staphylococcus aureus*, which generally colonizes human skin and mucosa^40^, was predominant in the male group. We found that an increase in *Staphylococcus aureus* was significantly associated with an increase of TEWL and decreased skin moisture levels in subjects. With *Staphylococcus aureus,* TEWL was reportedly higher and hydration level was lower in atopic dermatitis (AD) patients^41^, and low hydration was associated with high *S aureus* growth in AD patients^24^. Characteristically, in this study, the distribution of *Lactobacillus helveticus* was higher in women, which is similar to previous studies^38^. Several recent studies have investigated the beneficial effects of *Lactobacillus helveticus* fermentation within human skin epidermal cells^42,43^. Moreover, there have been cases in which *Lactobacillus helveticus* was orally administered as a probiotic to atopic patients to improve symptoms^36^, but its role as a human skin symbiotic bacteria has not yet been confirmed.

In conclusion, this study attempted to identify the microbiome on human facial skin in Korea through 16S rRNA analysis using next-generation sequencing analysis technology. We proved the age-related distribution of facial skin microbiome and the difference in the distribution of facial skin microbiome according to gender. We found out how these differences in facial skin microbiota distribution correlate with the skin's clinical characteristics. Correlation with clinical indicators in the skin microbiome at the species level was derived, and this approach is necessary to determine the balance of skin microbiome that we need to control for skin health. Nowadays, as various anti-aging cosmetics are on the market and various skincare industries develop, there can be a difference between actual age and biological skin age. It will be better to infer skin-improving microbials in consideration of the skin characteristics classified by sex hormones or men and women’s body characteristics^14^. Future studies must examine the facial skin microbiome and clinical skin parameter changes by considering the biological skin age and specifying cosmetics used in daily life.

## Supplementary Information

Supplementary Information 1.

Supplementary Information 2.

## Data Availability

The subject's information on this study and the results of the survey on subjects’ skin type and skin clinical characteristics are available as Supplementary Data 1 and 2, respectively. OTUs results and taxonomic composition results for each skin sample derived from 16S rRNA gene V3–V4 region sequencing are summarized in Supplementary Data. The dataset for the older and younger age groups are categorized for each skin site (forehead and cheek) and presented in Supplementary Data 3–6. The 16S rRNA gene sequencing data for all human-derived skin samples were registered as PRJNA723064 in the NCBI SRA (Sequence Read Archive) database.
