# Supplementary material for: Taxonomic profiling of skin microbiome and correlation with clinical skin parameters in healthy Koreans
Source: Sci Rep. 2021 Aug 11;11:16269. doi: 10.1038/s41598-021-95734-9 (PMC8358022; doi:10.1038/s41598-021-95734-9)
Supplement: Supplementary file 2 — Supplementary Information 2. [file 41598_2021_95734_MOESM2_ESM.pdf]

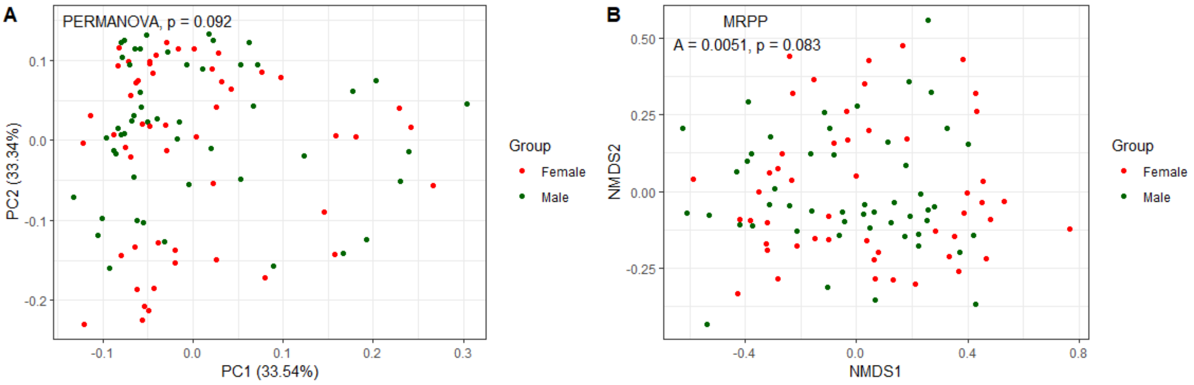

Supplementary Figure 1. Beta diversity between the female and male groups. (A) Principal Coordinates Analysis (PCoA) plot of UniFrac distances; (B) Non-metric multidimensional scaling (NMDS) plot of generalized UniFrac distances. Red circles represent the female group, and green circles represent the male group. P-values are displayed in the figures.

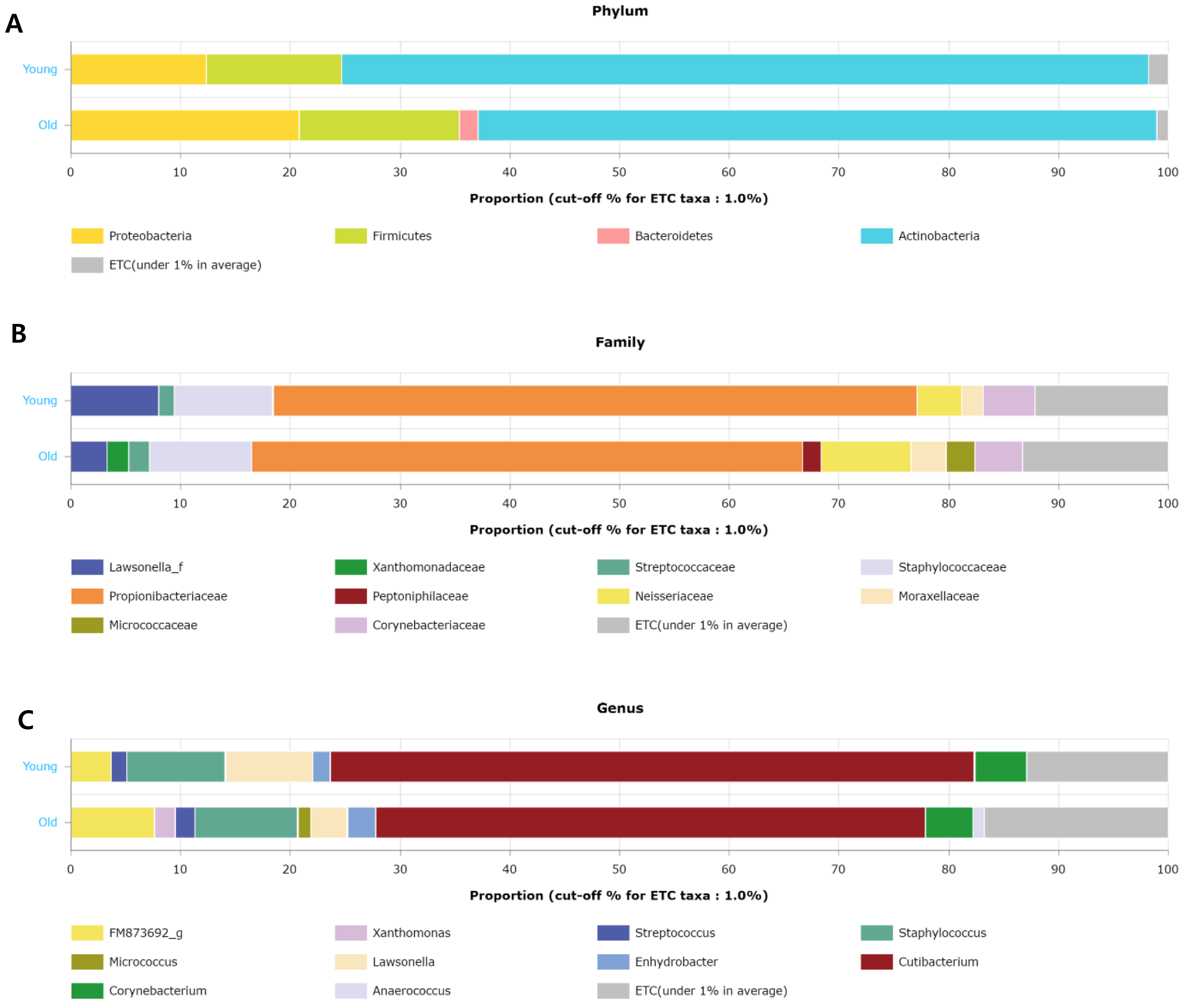

Supplementary Figure 2. The analysis results for all skin microbial composition between the younger (Young) and older (Old) groups (A), average relative abundances (%) of the four most abundant phyla; (B), average relative abundances (%) of the ten most abundant families; (C) average relative abundances (%) of the ten most abundant genera; Less than 1% abundant phylum are grouped and labeled as “ETC.”

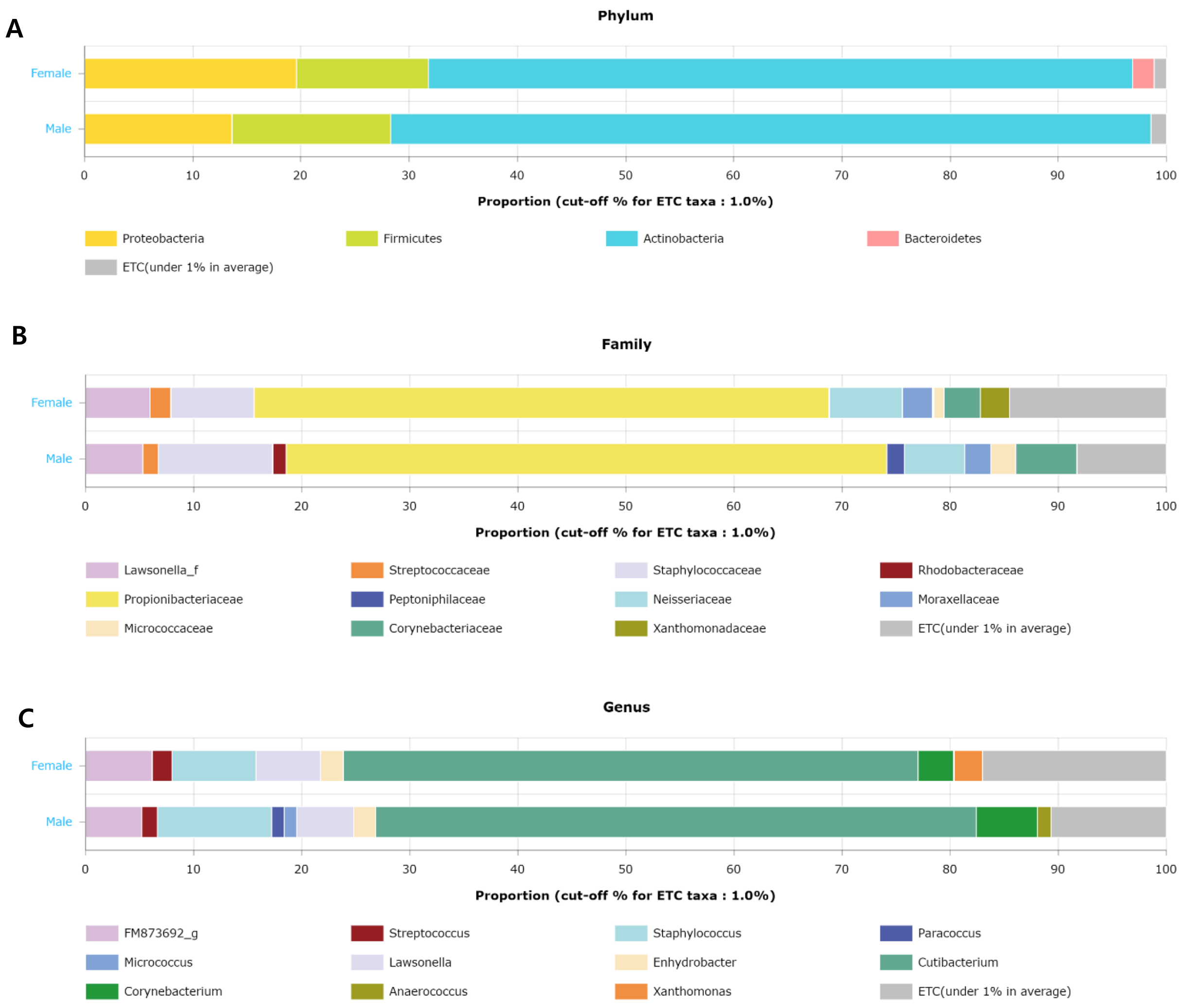

Supplementary Figure 3. The analysis results for all skin microbial composition between female and male (A), average relative abundances (%) of the four most abundant phyla; (B), average relative abundances (%) of the 11 most abundant families; (C) average relative abundances (%) of the 11 most abundant genera; Less than 1% abundant phylum are grouped and labeled as "ETC."

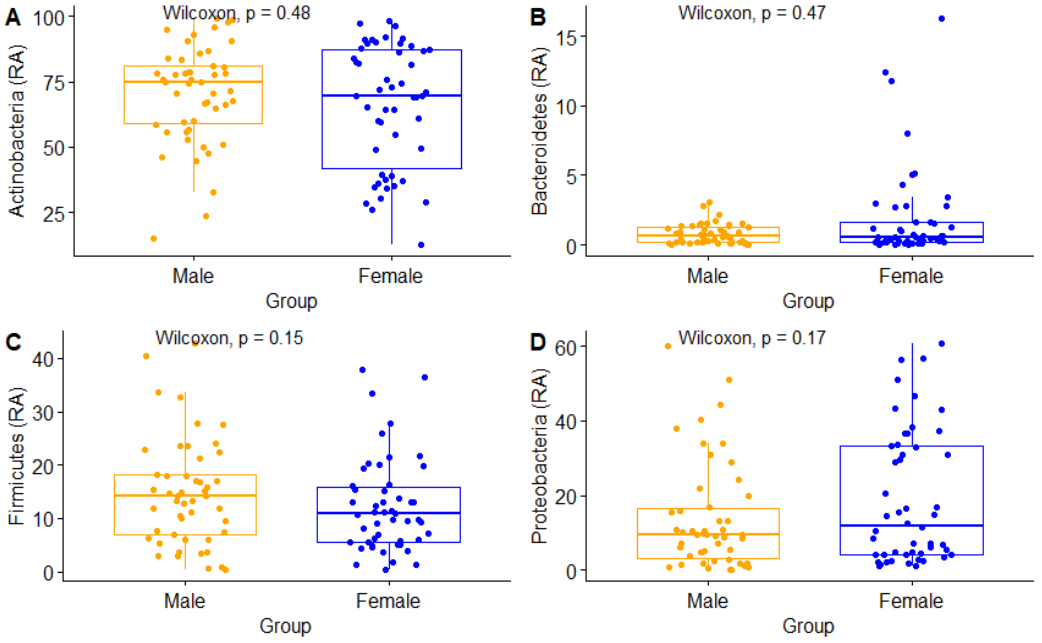

Supplementary Figure 4. Relative abundance (%) of four dominant phyla in skin samples between males and females. (A) Actinobacteria; (B) Bacteroidetes; (C) Firmicutes; (D) Proteobacteria. Wilcoxon signed-rank test was used for statistical analysis. P-values are displayed in the figures.

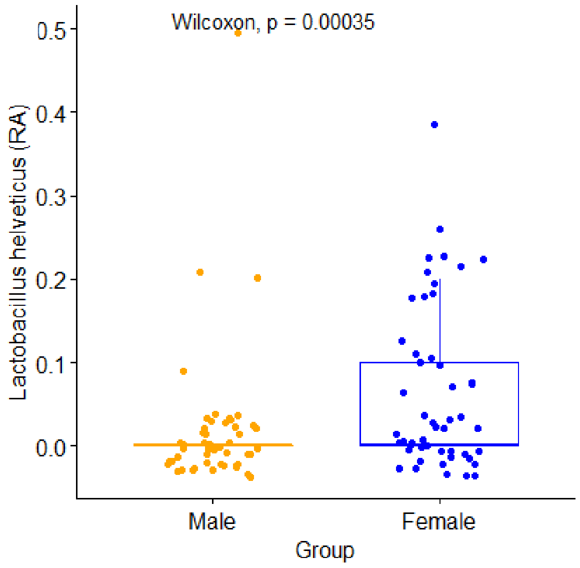

Supplementary Figure 5. Relative abundance (%) of *Lactobacillus helveticus* in skin samples between females and males. Wilcoxon signed-rank test was used for statistical analysis. P-value is displayed in the figure.
